# Do Seaducks Minimise the Flightless Period?: Inter- and Intra-Specific Comparisons of Remigial Moult

**DOI:** 10.1371/journal.pone.0107929

**Published:** 2014-09-24

**Authors:** Anouck Viain, Jean-Pierre L. Savard, Scott Gilliland, Matthew C. Perry, Magella Guillemette

**Affiliations:** 1 Département de Biologie, Chimie et Géographie, Université du Québec à Rimouski, Rimouski, QC, Canada; 2 Sciences and Technology, Environment Canada, Sainte-Foy, QC, Canada; 3 Canadian Wildlife Service, Environment Canada, Sackville, NB, Canada; 4 USGS-Patuxent Wildlife Research Center, Laurel, MD, United States of America; University of Zurich, Switzerland

## Abstract

Remigial moult is one of the crucial events in the annual life cycle of waterfowl as it is energetically costly, lasts several weeks, and is a period of high vulnerability due to flightlessness. In waterfowl, remigial moult can be considered as an energy-predation trade-off, meaning that heavier individuals would minimise the flightless period by increasing feather growth rate and energy expenditure. Alternatively, they could reduce body mass at the end of this period, thereby reducing wing-loading to increase flight capability. We studied timing of remigial moult, primary growth rates, flightlessness duration, and the pattern of body mass variation in 5 species of captive seaducks (*Melanitta fusca, M. perspicillata, Clangula hyemalis, Histrionicus histrionicus*, and *Somateria mollissima*) ranging in size from 0.5 to 2.0 kg. Their feather growth rates weakly increased with body mass (M^0.059^) and no correlation was found at the intra-specific level. Consequently, heavier seaduck species and especially heavier individuals had a longer flightless period. Although birds had access to food *ad libidum*, body mass first increased then decreased, the latter coinciding with maximum feather growth rate. Level of body mass when birds regained flight ability was similar to level observed at the beginning of remigial moult, suggesting they were not using a strategic reduction of body mass to reduce the flightlessness duration. We suggest that the moulting strategy of seaducks may be the result of a compromise between using an intense moult strategy (simultaneous moult) and a low feather growth rate without prejudice to feather quality. Despite the controlled captive status of the studied seaducks, all five species as well as both sexes within each species showed timing of moult reflecting that of wild birds, suggesting there is a genetic component acting to shape moult timing within wild birds.

## Introduction

During the annual cycle, numerous factors lead to the wear of feathers: mechanical abrasion induced by flight, reproduction and foraging activities [1], and degradation by ultraviolet light or by parasitic and bacterial infections [2], [3], [4]. As a result of flight feather degradation, flight performance is reduced [5], [6], [7]. Because feathers are somatic dead structures it is crucial for birds to replace periodically worn feathers during a process known as moult, which represents the major event of somatic production in the annual cycle of birds [8], [9].

Anatidae are among relatively few bird families that shed their flight feathers simultaneously once a year, rendering them flightless up to several weeks, with variations between species, age, and sex [10], [11]. Birds exhibiting simultaneous wing feather replacement are mainly aquatic, and function without flight, in contrast to sequential moulters, which replace a few flight feathers at a time. Simultaneous remigial moult in diving ducks is energetically costly [12], [13] comparatively to birds replacing flight feathers sequentially.

The length of the flightless period depends on the number of simultaneously growing feathers (moult intensity), the proportional length feathers when birds regain their flight ability, and the feather growth rate. Former investigations have shown that little variance exists in feather growth rate among birds ranging in size [14], [15]. Feather growth rate could be limited by an architectural constraint at the follicle level [16]. Recently, Rohwer *et al*. [16] demonstrated by allometric relationships that body mass increases much faster than feather growth rate, indicating that heavier species require more time to moult. A major implication of a low feather growth rate is that it could increase flightlessness duration for large simultaneous moulters. Thus, this would expose for example heavier diving birds to a higher predation risk and so it may be advantageous to minimise the flightless period [17], [18], [19], [20]. Here we replicate Rohwer *et al*. [16] work for a specific group of simultaneous moulters among the *Mergini*.

Flightless birds are subjected to different pressures leading to variation in flightlessness duration which is associated at benefits as well as at costs. First, flightless birds may be more vulnerable to predation [21], [22], [23], [24], [25] since the potential to escape predator attack is reduced. Some recent studies have shown that this stage could not be more unsafe than other stages of the annual cycle of birds [26], [27] nevertheless there are still few studies on this subject. Secondly, birds moulting at high latitude face to environmental constraints as onset of winter and freezing conditions, and so may have lower time to allocate to moult [28], [29]. Furthermore, it was recently shown that birds with less time to moult and with higher feather growth rate had poorer feather quality [30], [31], [32]. Finally, the capacity that birds have to meet nutritional and energy demands through exogenous and/or endogenous resources can also influence moult in terms of feather quality, timing and duration [33], [34], [35], [36]. Some authors have reported reduction of body mass during the flightless period of Anseriformes and have suggested that it was an adaptive strategy to reduce the duration of the flightless period [18], [19], [37]. Other authors have not reported any body weight loss during remigial moult [10], [38], [39], which contradicts that hypothesis [20], [37], [40], [41]. Therefore, we also tested the prediction that seaducks, especially heavier species, use strategic reduction of body mass to reduce the flightless period.

Using measurements of feather growth and body mass of 5 species of captive seaducks, the objectives of this study were to: 1) model and compare flight feather (9^th^ primary) growth rate between species and sexes, 2) compare the estimated timing and duration of flightlessness between species and between males and females, 3) test the hypothesis stipulating that heavier seaducks should have faster growth rate to minimise their flightless period, and 4) test the prediction that body mass reduction during remigial moult is associated with the regain of flight ability.

## Materials and Methods

### Ethics statement

All bird manipulations were approved by the Animal Care and Use Committee (ACUC, SOP No 007), for seaducks kept at the Patuxent Wildlife Research Center (Patuxent), and by the animal care committee of the Université du Québec à Rimouski (CPA-44-11-96), for Common Eiders kept at Rimouski. Eider eggs were collected under permit from Environment Canada - Canadian Wildlife Service (Permit Number: SC-24).

### Care of captive seaducks

All individuals of 4 species, 16 White-winged Scoters (*Melanitta fusca*, 9 males and 7 females, WWSC), 8 Surf Scoters (*M. perspicillata*, 5 males and 3 females, SUSC), 9 Long-tailed Ducks, (*Clangula hyemalis*, 3 males and 6 females, LTDU) and 19 Harlequin Ducks (*Histrionicus histrionicus*, 13 males and 6 females, HADU) were kept in captivity in monospecific groups. Eggs were collected with appropriate permits from the wild in Canada respectively from Redberry Lake (Saskatchewan), Lac Malbaie (Quebec), and Churchill (Manitoba), whereas HADU were obtained from a private propagator (Mr. A. Shouten) and were collected from the Olympic Peninsula (Washington). All eggs were hatched at the Patuxent Wildlife Research Center, Laurel, MD in USA, ducklings raised the same way, and were well acclimated to captivity. Birds were housed outside at Patuxent, and each pen, 4.3×8.6 m, contained a pool of fresh water with a diameter of 3.7 m and average depth of 0.5 m. Nine Common Eiders (*Somateria mollissima dresseri*, 4 males and 5 females COEI) were reared from eggs collected at Pointe-Métis, Quebec, with appropriate permits, in 2010. They were housed at Rimouski, Quebec in an indoor enclosure, 6.7×3.7 m with natural photoperiod and containing a pool, 6.0×1.7 m with continuously flowing fresh water of 0.4 m deep. Under these conditions eiders maintained their seasonal cycles of moult in synchrony with those of free-living individuals (AV, pers. obs.). All 5 species of seaducks were fed with Mazuri Sea duck Diet (#5681; 21.5% protein) *ad libitum* on a daily basis.

### Measurements from seaducks

All 4 species of captive seaducks kept at Patuxent were adults from 3 to 6 years old during this study. At a weekly interval from 8^th^ July to 8^th^ October 2008, all birds were caught with a landing net and held in a cat carrying case until being measured. The 9^th^ primary length (P9) was measured with a vernier caliper to the nearest millimetre, from the rim of the follicle to the distal tip of the feather. At 2-week intervals, birds were weighed (±1 g) with a digital scale. Measurements were made in a similar way on 2-year old Common Eiders between 27 July and 27 October 2012. Handling times were approximately 2–4 min per bird.

### Modelling feather growth

We tested 2 models commonly used for modelling biological growth: the logistic and Gompertz models. For our analyses, we retained the Gompertz, because it had the lower AIC_C_ score (Akaike's Information Criterion, corrected for small sample size [42]) and it was 5.76 times more support than the logistic model. For each bird of each species we estimated *A, μ* and *λ* as defined by the following function:
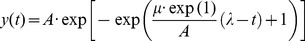
where *A* represents the asymptotic value which is an estimate of the maximum length of the feather, *μ* the maximum growth rate of P9, and *λ* the lag-phase. For each bird, we determined the delta growth rate between 5% and 90% of the final length of 9^th^ primary feather by calculated the change in P9 length divided by change in time. From each growth curve, the day of shedding of old P9 was estimated when feather reached artificially 1 mm long with Gompertz model (this value of 1 mm has been determined from shedding dates of old P9 noted in some of our captive individuals). Finally, the time between the day of shedding of old P9 and the day at which seaducks can fly again, estimated around 90% of P9 final length [43], was calculated for each bird to determine the flightlessness duration during moult.

### Modelling moult allometry

For simultaneous moulters, the delta growth rate of the longest flight feather (9^th^ primary) and its length, represent the best indicators of moult duration. Thus, to explore the time required to replace flight feathers in seaducks, we used the same scaling method as Rohwer *et al*. [16]. First, at the inter-specific level, we modeled the relationship between the length of the 9^th^ primary (mm) and the initial body mass (M in g). We also modeled the relationship between the delta growth rate of the 9^th^ primary (mm.day^−1^) and initial body mass. We used the allometric function *Υ = aM ^b^*, where “*a*” is a scaling constant and “*b*” the power of the relationship of *Υ* to mass. Our sample consisted of 5 species, but only 4 genera (*Clangula*, *Histrionicus*, *Melanitta*, and *Somateria*). The SUSC and WWSC are congener species and so could be a source of phylogeny mediated bias. We performed analysis of covariance on log-transformed values based on type III sums of squares with taxonomic genus entered as a main effect and we tested the interaction between genus and the independent variable. For all models the interactions were not significant, so the slopes of each genus could be modelled as one by the regression slope of models. However, regression slopes obtained with models did not differ from those determined by reduced major axis regression, so we used slopes obtained with simple regression throughout the paper. Finally, we also performed this procedure at the intra-specific level.

### Statistical analyses

Data were analyzed using R (R, v.3.0.1. Development Core Team, 2013). To model feather growth, we used the R package “*grofit*” (v.1.1, [44]). We performed linear mixed effects models fitted with maximum likelihood by using the R package “*nlme*” (v.3.1-111, [45]), with the interval of percent of feather length as the fixed effects and bird identity as the random effects. For normality, the Shapiro–Wilks test was used before proceeding with parametric tests and homogeneity of variance was tested with a Bartlett test. To compare weights along the remigial moult with initial body mass in the case of significant difference, we proceeded with a Dunnett post hoc comparison using the R package “*multcomp*” (v.1.2-19, [46]). We also used this package to proceed with Tukey's post hoc comparison to compare feather growth rate and flightlessness duration between species. To determine the power (“*b*”) of allometric relationships we compute major axis regression using the R package “lmodel2” (v.1.7-1, [47]). To test the idea that birds are losing body mass during remigial moult to reduce flightlessness, we subtracted the value of body mass reached at the phase in which birds are able to fly again from the initial body mass to obtain a delta for each individual of each species. We obtained, for each species and sex, the average delta for which we calculated a 95% CI (Confidence Interval) using the bootstrap method and 10,000 re-samplings. We verified the symmetry of the distribution by using the Skewness coefficient. Overall, the distribution was symmetric and we used the studentized bootstrap interval [48]. When the 95% CI excluded zero, the difference was significant at the 5% level, otherwise it was concluded that no meaningful difference existed. Differences between sexes were tested with Mann-Whitney test (*U*). All *p*-values were considered significant at the α = 0.05 level and means are given ± SD values throughout the paper.

## Results

Data for the present study will be available from the Dryad Digital Repository: doi:10.5061/dryad.p2kf0.

Body masses of 5 studied species ranged during early moult between 543±24 g and 1946±87 g, while the full length of 9^th^ primary varied between 129.0±3.3 mm and 181.1±5.5 mm (Table 1).

**Table 1:** Date of moulting, morphometric measurements and feather growth rate for 5 species of seaducks.

Species	Sex	n	Shedding day of old P9	Body mass (g)	P9 final length (mm)	Delta growth rate (mm.day^−1^)	Maximal growth rate (mm.day^−1^)
HADU	Female	6	13 August±3	543.3±24.2	129.0±3.3	3.43±0.10 *(c)*	4.66±0.13 (d)
	Male	13	7 August±5	609.2±29.0	132.1±3.6	3.48±0.14 *(c)*	4.77±0.22 (d)
LTDU	Female	6	1 August±12	563.3±25.8	137.7±1.7	4.20±0.13 *(a)*	5.91±0.21 (a)
	Male	3	24 July±8	610.0±85.4	142.0±4.0	4.14±0.28 *(a)*	5.86±0.38 (a)
SUSC	Female	3	22 July±6	756.0±54.1	147.7±0.6	3.71±0.07 *(b)*	5.18±0.12 *(c)*
	Male	5	13 July±11	848.0±31.1	154.2±2.6	3.64±0.10 *(b)*	5.11±0.23 *(c)*
WWSC	Female	7	2 August±4	1067.1±111.8	169.4±3.3	4.10±0.17 *(a)*	5.62±0.28 *(b)*
	Male	9	23 July±11	1270.0±93.8	178.1±3.0	4.03±0.12 *(a)*	5.55±0.19 *(b)*
COEI	Female	5	8 August±8	1905.6±115.2	180.5±5.6	3.82±0.17 *(b)*	5.39±0.25 *(b, c)*
	Male	4	28 July±7	1946.0±86.7	181.1±5.5	3.81±0.30 *(b)*	5.40±0.44 *(b, c)*

Mean shedding day of old 9^th^ primary ± SD, body mass (g) ± SD, final 9^th^ primary length (mm) ± SD, delta 9^th^ primary growth rate (mm.day^−1^) ± SD and maximal 9^th^ primary growth rate (mm.day^−1^) ± SD for 5 seaduck species (HADU, LTDU, SUSC, WWSC, and COEI). Species with the same letter are not significantly different and species with different letters are significantly different. Statistical results for delta 9^th^ primary growth rate (mm.day^−1^): Anova, F_4,56_ = 50.388, *p*<0.0001, Tukey's Post Hoc, with *p*<0.0001, *p*<0.0001, *p*<0.0001, *p* = 0.021, respectively for HADU vs. LTDU, HADU vs. WWSC, HADU vs. COEI and HADU vs. SUSC, with *p* = 0.369 for LTDU vs. WWSC and *p* = 0.268 for COEI vs. SUSC; for maximal 9^th^ primary growth rate: Anova, F_4,56_ = 50.289, *p*<0.0001, Tukey's Post Hoc, with *p*<0.0001, *p*<0.0001, *p*<0.001, *p* = 0.016, respectively for LTDU vs. HADU, LTDU vs. SUSC, LTDU vs. COEI, LTDU vs. WWSC and *p*<0.0001, *p*<0.0001, *p* = 0.001, respectively for HADU vs. WWSC, HADU vs. COEI and HADU vs. SUSC and with *p* = 0.323, *p* = 0.154 and *p*<0.001, respectively for COEI vs. SUSC, COEI vs. WWSC and SUSC vs. WWSC.

### Timing of initiation of remigial moult

**Figure 1:**
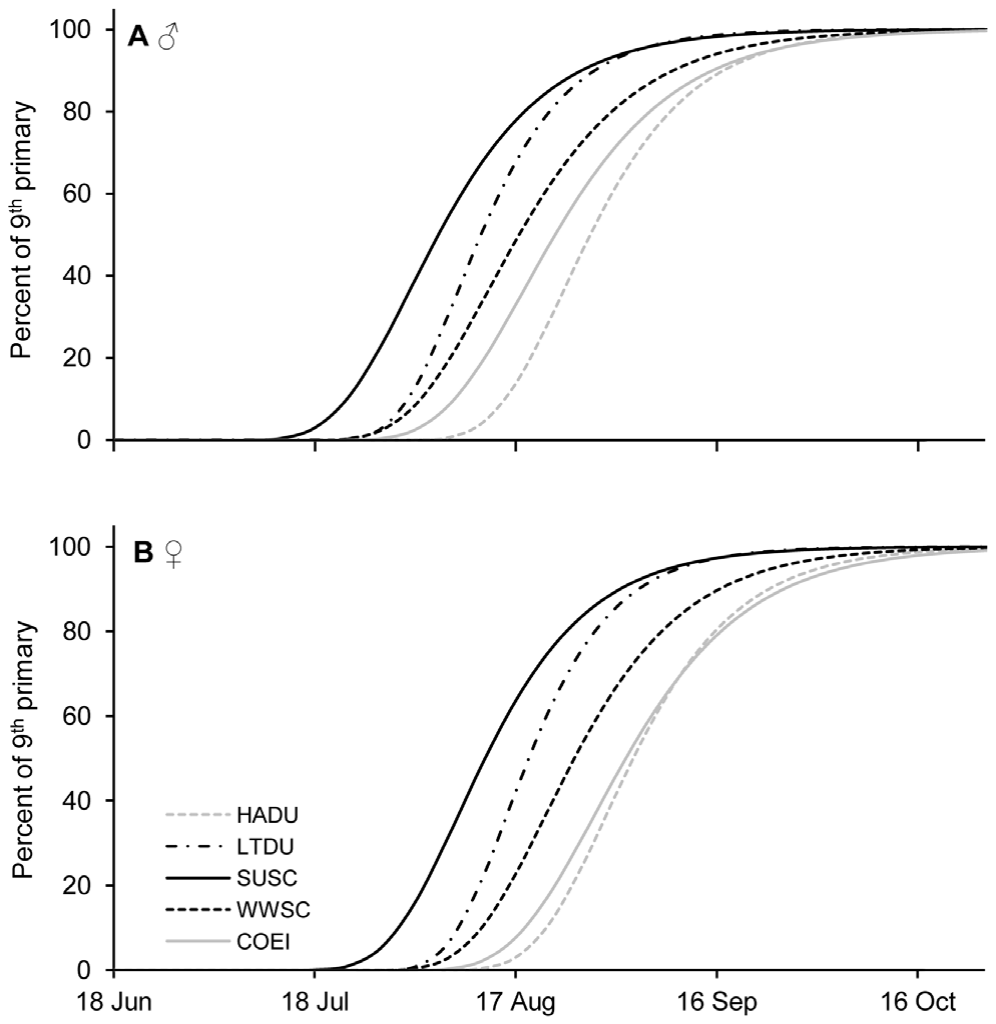
Feather growth rates of the 5 seaduck species. Gompertz 9^th^ primary growth models for 5 seaduck species; Harlequin Ducks (*Histrionicus histrionicus*, HADU), Long-tailed Ducks (*Clangula hyemalis*, LTDU), Surf Scoters (*Melanitta perspicillata*, SUSC), White-winged Scoters (*Melanitta fusca*, WWSC) and Common Eiders (*Somateria mollissima dresseri*, COEI) with (**A**) representing males and (**B**) females.

Males of the 5 species initiated remigial moult significantly earlier than females (*U* = 319, *p* = 0.042; Table 1). Interspecific patterns were similar between sexes (Fig. 1): SUSC was the first species to moult primaries, with a mean shedding date of P9 at 13 and 22 July respectively for males and females (Table 1); LTDU was the second species to moult, with a shedding date at 24 July and 1 August, respectively for males and females; WWSC moult almost at the same date than LTDU; COEI was the fourth species to moult followed by HADU, which moulted in August.

### Inter-specific comparison of feather growth rates

Among the 5 seaduck species, the maximal growth rate of P9 ranged between 4.66±0.13 and 5.91±0.21 mm.day^−1^, which represents 3.6% and 4.3% of daily percent change in remiges (Table 1), respectively for females of HADU and females of LTDU, while the delta growth rate ranged between 3.43±0.10 and 4.20±0.13 mm.day^−1^ (Table 1). The daily percent change in feather length over the period of 5% to 90% of feather development for the 5 species varied between 2.1% and 3.1%. The maximal feather growth rate of LTDU was faster than the maximal feather growth rate of the 4 other species, while HADU had the slowest (see statistical details in the legend of Table 1). COEI had a similar maximal feather growth rate as the 2 scoter species, while WWSC and SUSC had different maximal growth rates (Table 1). HADU had a slower delta growth rate than the 4 other species (see statistical details in legend of Table 1). The feather delta growth rates of LTDU and WWSC were the fastest and similar (Table 1). It was similar for COEI and SUSC and slower than delta growth rates of LTDU and WWSC (Table 1). For each species, maximal and delta growth rates of P9 did not differ between females and males (*U* = 48.00, *p* = 0.430; *U* = 7.00, *p* = 0.606; *U* = 4.00, *p* = 0.297; *U* = 26.00, *p* = 0.560 and *U* = 11.00, *p* = 0.806, respectively for HADU, LTDU, SUSC, WWSC, and COEI).

### Moult allometries

**Figure 2:**
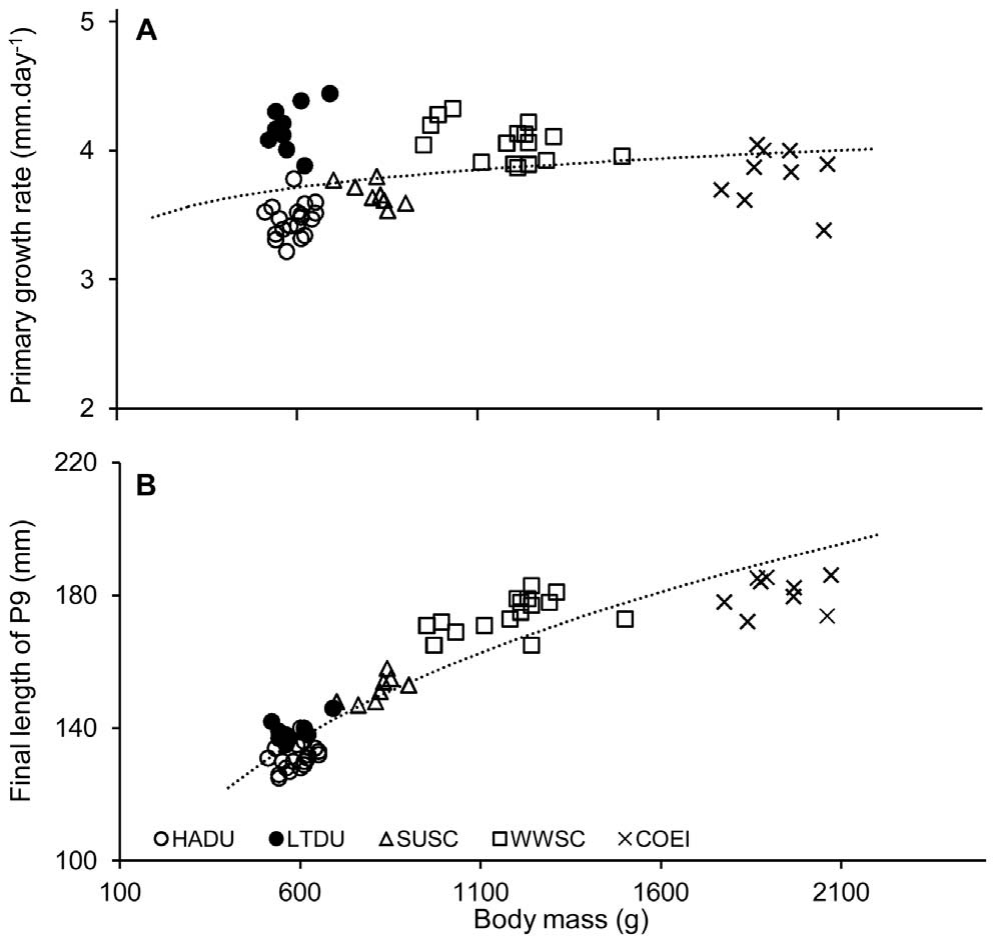
Relationships between body mass and primary growth rate, and P9 length in 5 seaduck species. Allometric relationships among 5 seaduck species between: (**A**) body mass (g) and delta 9^th^ primary growth rate (mm.day^−1^), (**B**) body mass (g) and final length of 9^th^ primary (mm). Dotted line represents the allometric relation for the 5 species (HADU, LTDU, SUSC, WWSC and COEI) (**A**) allometric relation: M^0.059^, (**B**) allometric relation: M^0.285^.

Among the 5 species, average daily delta growth rate of P9 scaled to body mass as M^0.059^ (Fig. 2A; *r* = 0.305, *p* = 0.017, 59 d.f. and 95% CI 0.011–0.107) and the terminal length of the 9^th^ primary scaled to body mass as M^0.285^ (Fig. 2B, *r* = 0.931, *p*<0.0001 and 95% CI 0.256–0.314). As these species undergo a simultaneous replacement of the primaries, the time required to replace all flight feathers increases as 0.226 the power of the body mass (M^0.285^/M^0.059^ = M^0.226^). At the intra-specific level, no correlations were found between feather delta growth rate and body mass (*r* = 0.200, *p* = 0.415 for HADU, *r* = 0.321, *p* = 0.399 for LTDU, *r* = 0.683, *p* = 0.062 for SUSC, *r* = 0.459, *p* = 0.074 for WWSC and *r* = 0.141, *p* = 0.717 for COEI). However, there was a hint of a possible relationship in SUSC and WWSC if we used the α = 0.10 level.

### Duration of estimated flightless period

**Figure 3:**
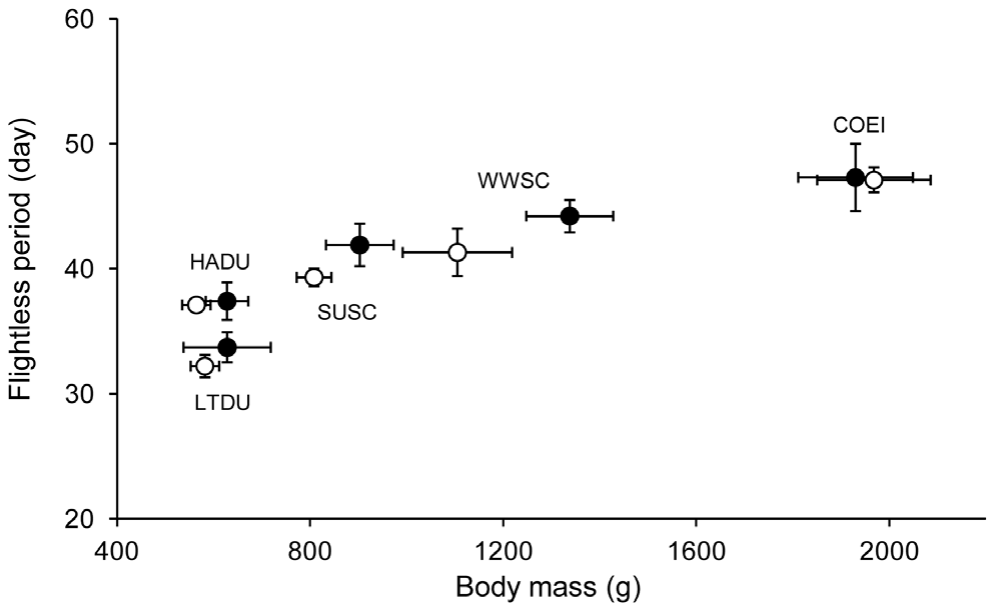
Relationship between body mass of 5 seaduck species and flightless period. Representation of the flightless period (days) according to the body mass for the 5 seaduck species (HADU, LTDU, SUSC, WWSC and COEI). Females are represented by white circles and males by black circles.

The estimated flightlessness duration varied among the 5 seaduck species (Anova, F_4,56_ = 104.584, *p*<0.0001). However, flightlessness duration did not differ between sexes except for the WWSC (*U* = 56, *p* = 0.010), which was also the species showing the greatest sexual dimorphism at the level of 9^th^ primary length and body mass. The shortest flightless period was recorded for LTDU with 32.2±0.9 and 33.7±1.3 days respectively for females and males (Fig. 3). COEI had the longest estimated flightless period with 47.1±1.0 and 47.3±2.7 days respectively, for females and males. HADU had an estimated flightlessness duration around 37 days (37.1±0.4 and 37.4±1.5, respectively for females and males). Scoters had similar estimated flightless periods (Tukey's Post Hoc, *p* = 0.067). SUSC were flightless for 39.3±0.7 and 41.9±1.7 days, respectively, for females and males. Females of WWSC were flightless for 41.3±1.9 days, while males were flightless for 44.2±1.3 days.

### Variation of body mass during flight feather moult

**Figure 4:**
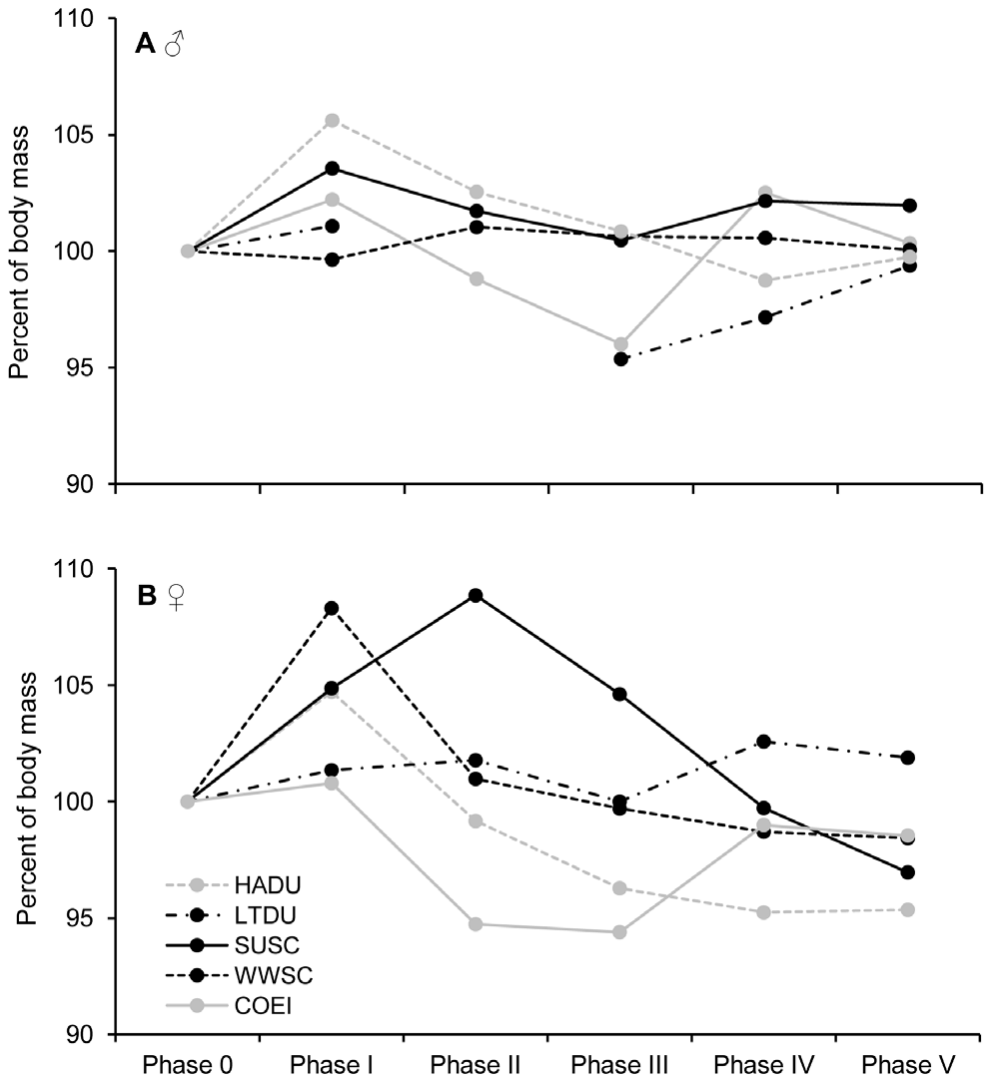
Inter-specific body mass variation during remigial moult. Changes in mean body mass of birds (as percentage of initial weight) based on the percentage growth of the 9^th^ primary for 5 seaduck species; Harlequin Ducks (HADU), Long-tailed Ducks (LTDU), Surf Scoters (SUSC), White-winged Scoters (WWSC) and Common Eiders (COEI) with (**A**) representing males and (**B**) females. *Phase 0* corresponds to the value of initial weight before the shedding of flight feathers, *Phase I* represents feather emergence and the start of feather growth, *Phase II* corresponds to the period of fast feather growth rate and where maximal growth rate is reached, *Phase III* is the period of the first slowing down of growth rate, *Phase IV* corresponds to the second slowing down of growth rate and the period during which birds are able to fly again, *Phase V* is the end of the remigial moult when final length of P9 is reached.

**Figure 5:**
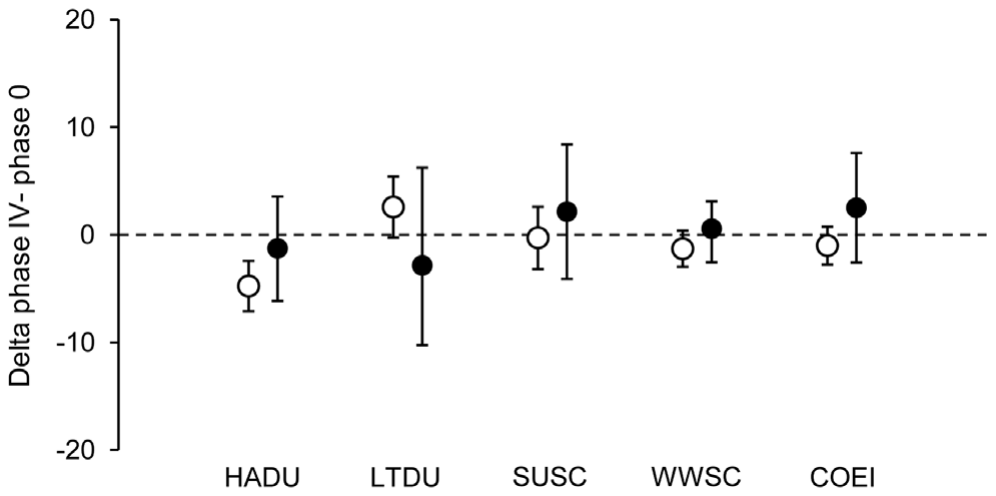
Intra-specific body mass variation for the studied species. Average deltas, representing the difference between body mass recorded at the phase IV of the moult in which seaducks are able to re-fly and initial body mass, and their respective confidence intervals. When the confidence intervals exclude the zero line, the delta is declared significant at 5% level (see Materials and methods).

During remigial moult, body mass of both sexes tented to increase at the beginning of moult after which it declined until it stabilized around initial body mass at the end of the moult. This pattern was more pronounced in females than males (Fig. 4.). The increase of body mass at the beginning of moult was significant for both sexes in HADU (F_5,20_ = 12.075, *p*<0.001, Dunnett's test *p* = 0.013 and F_5,44_ = 2.823, *p* = 0.027, Dunnett's test *p*<0.001, respectively for females and males) and for female scoters (F_5,7_ = 11.816, *p* = 0.003, *p*<0.001 and *p* = 0.021, Dunnett's test *p* = 0.038 and F_5,21_<0.001, Dunnett's test *p*<0.001, respectively for SUSC and WWSC). During remigial moult, female COEIs and HADUs were the only species/sex cohorts that reached a lighter body mass than the initial value (F_5,38_ = 4.61, *p* = 0.002, Dunnett's test *p* = 0.025 and F_5,20_ = 12.075, *p*<0.001, Dunnett's test *p* = 0.031, respectively). Female HADUs were the only cohort that was significantly lighter at the end of the moult (*p* = 0.002 at the maximal 9^th^ primary feather length, phase V). Finally, to test the hypothesis that birds lose body mass during remigial moult to shorten flightlessness, we also compared the initial body mass with body mass reached at phase IV, when birds are able to fly again. The mean body mass reached at the phase IV was not lighter than the mean initial body mass except for females of HADU (Fig. 5).

## Discussion

### Applicability to wild seaducks

In this study, the results obtained for the timing, flightlessness duration, and growth rates of remigial moult were comparable to those obtained on wild birds. Indeed, our data that the timing of remigial moult differ among species and sexes, with an initiation of remigial moult earlier in males than females, has been observed in most wild waterfowl species [10], [43], [49], [50], [51], [52]. The initiation dates of remigial moult for males of the 5 studied species are similar to dates reported in the wild [43], [50], [53], [54], [55], [56], [57]. In contrast, for captive females the mean shedding dates were earlier than their wild counterparts. As a consequence, differences between sexes are in average only of 6 to 11 days in our study while, depending on species, it is about 3 weeks in the wild [49], [51], [54], [58], [59], [60]. We thus suggest that the differences in the start of moult, observed between captive and wild females, are related to their status of non-breeders in our study as none were in a breeding state before they started moulting. For instance, there is evidence that moult starts later in breeding birds in the wild [5], [10], the mechanism here being that elevated level of prolactin associated with parental care inhibits the onset of moult [61]. Indeed, it is well known that sex hormones but also environmental conditions (light levels, temperature, food availability, etc.) are factors acting in moult timing [62], [63], [5]. Interestingly, among the 5 studied species of seaducks, and despite the non-breeding status of birds, and controlled environment due to captivity, females still moulted later than males. These results underline a potential involvement of genetic/sex-specific factor in the launching of moult mechanisms. Such a result would not be possible to obtain in natural wild settings, and we are unaware of other studies clearly showing a sex-specific genetic component shaping moult timing after environmental factors have been controlled. The interaction between genetic and environmental effects on moult strategies in birds disserves further investigation.

The estimates of flightlessness duration, based on the Gompertz growth model and the 90% of P9 final length generate results comparable to values from the literature. However, differences are observed for HADU [50] and COEI [12]. Indeed, Robertson *et al*. [50] used the value of 70% of P9 final length reported by Hohman *et al*. [10] to estimate HADU flightless period, while it has been estimated to be higher for most seaduck species [43], [51], [64] (around 80% for male SUSC, SG, pers. obs.). For COEI, Guillemette *et al*. [12] used a behavioural technic to estimate flightlessness duration, which may have caused the discrepancy. Again, the difference may also stem from a general value (90%) applied to all species, while it was observed to be lower for COEI (AV, pers. obs.).

The delta primary growth rates for each studied species were consistent with previous results on wild birds [43], [50], [26]. Similarly, the absence of difference in feather growth rates found between sexes conforms to former investigations [10]. However, no data relating to primary growth rates of COEI and LTDU were available. For COEI, our estimates of delta and maximal primary growth rates represent respectively, 2.1% and 3.0% daily change in length, which fall within the range of daily change values found for most waterfowl species (2–3%, [10]). LTDU presented higher values (respectively 3.1% and 4.3% for females and 2.9% and 4.1% for males). Among all five species, LTDU breeds at the furthest north [65] while HADU, the slowest moulter in this study, is the southern-most species [52]. Thus, the timing constraint and weather factors at this latitude might be important factors for a rapid moult as it is the case for others birds such the White-crowned sparrow (*Zonotrichia leucophrys nuttalli and Z. l. Pugetensis*) [29], shorebirds [66], [52], and the least Auklets (*Aethia Pusilla*) [28]. As with our sex-specific results, the finding that moult timing of our non-breeding captive birds reflected that of each species in the wild suggests a genetic component interacting with environmental factors (such as light-level regimes at different latitudes, food resources, and climatic conditions found under natural conditions) to shape moult timing.

### Allometry of feathers growth

Our study is based on the assumption that a flightless state is under numerous constraints (predation risk, temporal constraints, food availability etc). Thus, it would be advantageous for birds to minimise the flightless period by increasing feather growth rate. In the present study, we showed that heavier seaduck species required more time to grow their flight remiges, which qualitatively supports the findings of Rohwer *et al*. [16]. More specifically, primary growth rate scales as M^0.059^, which is 3 times lower than the allometric exponent derived by Rohwer *et al*. [16] for birds in general (M^0.171^). Indeed, doubling the body mass in our allometric relationship only results in an increase of 4.0% growth rate against 12.6% for Rohwer's *et al*. [16] study. Now, using the length of the longest primary, these authors reported 2 power estimates for their allometric relationship relating feather length and body mass (one in result section and a different one in the methods section). Using the raw data of Rohwer *et al*. [16], we validated the result found in their methods section where the longest primary feather increases as the power of 0.325 of body mass (95% CI 0.302–0.346), which is close but higher to our result (M^0.285^ with 95% CI, 0.256–0.314). Thus, the time required to complete moult increases as the power 0.154 of body mass [16] while it is higher for *Mergini* with M^0.226^. As a result, flightlessness duration increases with body mass in seaducks (Fig. 3).

At the intra-specific level and for each of the 5 species studied, no significant correlation between feather growth rate and body mass was found at the α = 0.05 level but there were marginally significant correlations for SUSC and WWSC. Clearly larger sample sizes are needed to determine whether scoters differ from other species on that point. The absence of relationship at the intra-specific level and the disproportionate (slow) increase of feather growth rate in relation to body mass at the inter-specific level may reflect the physiological and structural limits at which feathers can be produced [14], [15], [16]. Thus, our results support the original hypothesis of Prevost [14] stating that it is mainly Anseriformes that may have reached the limit of feather growth rates. Based on allometric principles, Rohwer *et al*. [16] deduced that structural constraints at the level of the cylindrical growing region of primary feathers might lead to a physiological bottleneck. More specifically, the diameter of the follicle and the number of rows of dividing cells could limit feather growth rate of large birds and could increase the flightlessness duration for simultaneous moulters (Fig. 3). This may also be the result of a trade-off between the use of a fast moult strategy (simultaneous moulters vs. sequential moulters) and feather quality. Indeed, some recent studies have shown that fast moulters had lower feather quality compared to slow moulters, in terms of mass, coloration, and physical proprieties of the feather [30], [31], [32], [67], [68], [69].

### Variations in body mass during remigial moult

Wing-loading of an individual is an important parameter that influences takeoff and ability to escape successfully from a predator attack [70], [71], [34], [72]. Captive seaducks of the 5 species studied showed variations in their body mass during remigial moult despite that they had access to food *ad libidum*. The range and pattern of body variations differ between species, sex, and size. We interpret the marked increase of body mass 15 days after the primaries were shed (Phase I), followed by a decrease of body mass (Phase II), as a strategy to allocate body reserves to a period coinciding with the highest rate of feather growth (Phase II, Fig 4). In other words, the decrease of body mass of phase II corresponds to the steep slope of feather growth models, hence a period of high energy demand. Similar observations of body mass loss during remigial moult were recorded for different species of waterfowl in both captive and wild situations [10], [17], [64], [73], [74]. However, the SUSC and WWSC male scoters did not show any significant variation of body mass in our study, as observed for Common Scoters (*Melanitta nigra*) [39].

One pervasive idea in the literature is that body mass loss observed during the remigial moult may be an adaptive strategy to reduce flightlessness duration in a way, that lighter birds will accelerate the regain of flight ability [17], [75]. Comparing body mass at regain of flight ability (phase IV) with initial body mass (phase 0), we observed a trend for lower body mass of females at phase IV. In contrast, we observed an opposite trend for males. Nevertheless, most studied species, excepted females of HADU, did not have a lighter body mass at regain of flight ability (between 80% to 100% of final 9^th^ primary length, Fig. 5). Thus, our results under controlled environment (constant food supply, no predation risks, etc.) do not support the idea of a strategic body mass management that would reduce flightlessness duration in these species. Variations in body mass observed in wild birds, such the reduction in mass of moulting male LTDU in the Beaufort Sea [76] could simply result from a depletion of their food since birds congregate and exploit a smaller foraging habitat. Ouellet *et al*. [77] reviewed the various mechanisms that could increase lift during takeoff of birds and, apart wing-loading reduction, they pin-point to (1) the enhancement of mechanical power with larger pectoral muscles and (2) improvement of muscle metabolic output with increased enzyme activity. Several studies have documented changes in the mass of legs and flight muscle mass in waterfowl with muscle mass decreasing at the beginning of remigial moult and increasing towards the end [20], [39], [76]. Such a level of organ flexibility might enhance flight capability despite the fact that body mass remains constant. Further studies should investigate these potential strategies in moulting birds.

In conclusion, our results concerning timing and flightlessness duration are similar to various published studies although we did not find any support for the idea that body mass reduction at the end of remigial moult is used to minimise the flightless period. In addition, our power estimate of the allometric relationship relating body mass and feather growth rate is lower than birds in general, indicating that seaducks do not use a high feather growth rate to minimise flightlessness duration. Further investigations should seek potential explanations for that observation in terms of physiological or structural constraint, although we contend that the relationship between feather quality and feather growth rate seems to be the most potential avenue to explain such a difference. Finally, our sex-specific results of timing of moult observed in a controlled environment and reflecting that of wild seaducks, suggest that there is a genetic component acting to shape moult timing within wild birds, in addition to well-known environmental effects.

## References

[1] Weber TP, Borgudd J, Hedenström A, Persson K, Sandberg G (2005) Resistance of flight feathers to mechanical fatigue covaries with moult strategy in two warbler species. Biol Lett 1: 27–30. 17148120 10.1098/rsbl.2004.0244 PMC1629048

[2] Bergman G (1982) Why are the wings of *Larus f. fuscus* so dark? Ornis Fenn 59: 77–83.

[3] Burt EH Jr, Ichida JM (1999) Occurrence of feather-degrading bacilli in the plumage of birds. Auk 116(2): 364–372.

[4] Figuerolaa J, Domenech J, Senar JC (2003) Plumage colour is related to ectosymbiont load during moult in the serin, *Serinus serinus*: an experimental study. Anim Behav 65: 551–557.

[5] Ginn HB, Melville DS (1983) Moult in Birds. BTO Guide 19. Tring: British Trust for Ornithology. 112 p.

[6] Tucker VA (1991) The effect of molting on the gliding performance of a Harris' hawk (*Parabuteo unicinctus*). Auk 108(1): 108–113.

[7] Murphy ME, King JR (1992) Energy and nutrient use during moult by White-crowned sparrows *Zonotrichia leucophrys gambelii* . Ornis Scand 23(3): 304–313.

[8] Walsberg GE (1983) Avian ecological energetics. In: Farner DS, King JK, Parkers KC, editors, Avian biology. New York: Academic Press. pp. 161–220.

[9] Guillemette M, Butler PJ (2012) Seasonal variation in energy expenditure is not related to activity level or water temperature in a large diving bird. J Exp Bio 215: 3161–3168 10.1242/jeb.061119 22660783

[10] Hohman WL, Ankney CD, Gordon DH (1992) Ecology and management of postbreeding waterfowl. In: Batt BDJ, Afton AD, Anderson MG, Ankney CD, Johnson DH, Kadlec JA, Krapu GL, editors. Ecology and Management of Breeding Waterfowl. Minneapolis. University of Minnesota Press. pp. 128–189.

[11] Fox AD, Flint PL, Hohman WL, Savard J-PL (2014) Waterfowl habitat use and selection during the remigial moult period in the northern hemisphere. Wildfowl Special issue *in press*.

[12] Guillemette M, Pelletier D, Grandbois JM, Butler PJ (2007) Flightlessness and the energetic cost of wing molt in a large sea duck. Ecology 88(11): 2936–2945. 18051662 10.1890/06-1751.1

[13] Portugal SJ, Isaac R, Quinton KL, Reynolds S (2009) Do captive waterfowl alter their behaviour patterns during their flightless period of moult? J Ornithol 151(2): 443–448.

[14] Prevost Y (1983) The moult of the Osprey *Pandion haliaetus* . Ardea 71: 199–209.

[15] Langston NE, Rohwer S (1996) Molt–breeding tradeoffs in albatrosses: life history implications for big birds. Oikos 76: 498–510.

[16] Rohwer S, Ricklefs RE, Rohwer VG, Copple MM (2009) Allometry of the Duration of Flight Feather Molt in Birds. PLoS Biol 7(6): e1000132 10.1371/journal.pbio.1000132 19529759 PMC2690433

[17] Douthwaite RJ (1976) Weight changes and wing moult in the red-billed teal. Wildfowl 27: 123–127.

[18] Owen M, Ogilvie MA (1979) Wing molt and weights of barnacle geese in Spitsbergen. Condor 81: 42–52.

[19] Pehrsson O (1987) Effects of body condition on molting in Mallards. Condor 89(2): 329–339.

[20] Fox AD, Kahlert J (2005) Changes in body mass and organ size during wing moult in non-breeding greylag geese *Anser anser* . J Avian Biol 36: 538–548.

[21] Booth CJ, Ellis P (2006) Common eiders and common guillemots taken by killer whales. Br Birds 99: 533–535.

[22] Guillemette M, Ouellet J-F (2005) Temporary flightlessness as a potential cost of reproduction in pre-laying Common Eiders Somateria mollissima. Ibis 147: 301–306.

[23] Moore PG (2001) Concerning grey seals killing eider ducks in the Clyde Sea area. J Mar Biolog Assoc 81: 1067–1068.

[24] Morgan R (1986) Eider attacked by grey seal. Br. Birds 79: 338.

[25] Smith WE (2006) Moulting common eiders being devoured by killer whales. Br. Birds 99: 264.

[26] Iverson S, Esler D (2007) Survival of female Harlequin Ducks during wing molt. J Wild Manage 71(4): 1220–1224.

[27] Hogan D, Thompson JE, Esler DE (2013) Survival of Barrow's Goldeneyes during remigial molt and fall staging. J Wild Manage 77: 701–706.

[28] Bond AL, Konyukhov NB, Jones IL (2013) Variation in primary molt in the least auklet. Condor 115(2): 348–355.

[29] Mewaldt LR, King JR (1979) Latitudinal variation of postnuptial molt in pacific coast White-crowned sparrows. Auk 95: 168–179.

[30] Dawson A, Hinsley SA, Ferns PN, Bonser RHC, Eccleston L (2000) Rate of moult affects feather quality: a mechanism linking current reproductive effort to future survival. Proc R Soc B 267(1457): 2093–2098. 10.1098/rspb.2000.1254 PMC1690790 11416914

[31] Serra L, Griggio M, Licheri D, Pilastro A (2007) Moult speed constrains the expression of a carotenoid-based sexual ornament. J Evol Biol 20(5): 2028–2034. 17714319 10.1111/j.1420-9101.2007.01360.x

[32] De La Hera I, Pérez-Tris J, Tellería JL (2009) Migratory behaviour affects the trade-off between feather growth rate and feather quality in a passerine bird. Biol J Linn Soc Lond 97: 98–105.

[33] Swaddle JP, Witter MS (1997) The effects of molt on the flight performance, body mass, and behavior of European Starlings (*Sturnus vulgaris*): an experimental approach. Can J Zool 75: 1135–1146.

[34] Murphy ME, King JR (1987) Discrimination by molting White-crowned sparrows given diets differing only in sulfur amino acid concentration. Physiol Zool 60(2): 279–289.

[35] Murphy ME, King JR, Lu J (1988) Malnutrition during the postnuptial molt of White-crowned Sparrows: feather growth and quality. Can J Zool 66: 1403–1413.

[36] Catry P, Poisbleau M, Lecoq M, Phillips RA (2013) Differences in the timing and extent of annual moult of black-browed albatrosses Thalassarche melanophris living in contrasting environments. Polar Biol 36(6): 837–842.

[37] Ankney CD (1979) Does the wing molt cause nutritional stress in lesser snow geese? Auk 96: 68–72.

[38] Fox AD, Kahlert J, Walsh AJ, Stroud DA, Mitchell C, et al (1998) Patterns of body mass change during moult in three different goose populations. Wildfowl 49: 45–56.

[39] Fox AD, Hartmann P, Petersen IK (2008) Body mass and organ size change during wing moult in common scoter. J Avian Biol 39: 35–40.

[40] King JR, Murphy ME (1985) Periods of nutritional stress in the annual cycles of endotherms: fact or fiction? Am Zool 25(4): 955–964.

[41] Brown CR, Bryant DM (1996) Energy expenditure during molt in dippers (*Cinclus cinclus*): no evidence of elevated costs. Physiol Zool 69(5): 1036–1056.

[42] Motulsky H, Christopoulos A (2004) Fitting models to biological data using linear and non-linear regression: a practical guide to curve fitting. Oxford University Press, Oxford, 352p.

[43] Dickson RD, Esler D, Hupp JW, Anderson EM, Evenson JR, et al (2012) Phenology and duration of remigial moult in Surf Scoters (*Melanitta perspicillata*) and White-winged Scoters (*Melanitta fusca*) on the Pacific coast of North America. Can J Zool 90: 932–944.

[44] Kahm M, Hasenbrink G, Lichtenberg-Frate H, Ludwig J, Kschischo M (2010) Grofit: fitting biological growth curves with R. Journal of Statistical Software. 33(7): 1–21 Available: http://www.jstatsoft.org/v33/i07/ Accessed 2013 April 15..

[45] Pinheiro J, Bates D, DebRoy S, Sarkar D, the R Development Core Team 2013 nlme: Linear and Nonlinear Mixed Effects Models. R package version 3.1-111. Available: http://CRAN.R-project.org/package=nlme. Accessed 2013 April 15.

[46] Hothorn T, Bretz F, Westfall P (2008) Simultaneous Inference in General Parametric Models. Biom J 50(3): 346–363. 18481363 10.1002/bimj.200810425

[47] Legendre P (2011) lmodel2: Model II Regression. R package version 1.7-1/r1794. Available: http://R-Forge.R-project.org/projects/vegan/. Accessed 2013 April 15.

[48] Good P (2006) Resampling Methods - A Practical Guide to Data Analysis, (3^rd.^ edition). Boston, Basel and Berlin. Birkhäuser. 218 p.

[49] Petersen M (1981) Populations, feeding ecology and molt of Steller's Eider. Condor 83: 256–262.

[50] Robertson GJ, Cooke F, Goudie RI, Boyd WS (1997) The timing of arrival and molt chronology of Harlequin Ducks *Histrionicus histrionicus* . Wildfowl 48: 147–155.

[51] Hogan D (2012) Remigial Molt Phenology and Remigial Growth Rates of Barrow's Goldeneyes in northwestern Alberta. M.Sc. thesis, Department of Biological Sciences, Simon Fraser University, Burnaby, B.C.

[52] Pyle P (2008) Identification Guide to North American Birds, Part 2. Slate Creek Press, Point Reyes Station, California, USA.

[53] Salomonsen F (1968) The moult migration. Wildfowl 19: 5–24.

[54] Joensen AH (1973) Moult migration and wing-feather moult of seaducks in Denmark. Danish Rev Game Biol 8(4): 1–42.

[55] Jepsen PU (1984) Observations of moulting Eider and breeding Common Eider *Somateria mollissima* at Nordaustlandet, Svalbard, in 1979. Polar Res 2 *n.s*.: 19–25.

[56] Adams PA, Robertson GJ, Jones IL (2000) Time-activity budgets of Harlequin ducks molting in the Gannet Islands, Labrador. Condor 102(3): 703–708.

[57] Petersen MR, Mccaffery BJ, Flint PL (2003) Post-breeding distribution of Long-tailed Ducks *Clangula hyemalis* from the Yukon-Kuskokwim Delta, Alaska. Wildfowl 54: 103–113.

[58] Frimer O (1994) Autumn arrival and moult in King eiders (*Somateria spectabilis*) at Disko, West Greenland. Arctic 47(2): 137–141.

[59] Mosbech A. Boertmann D (1999) Distribution, Abundance and Reaction to Aerial Surveys of Post-breeding King Eiders (*Somateria spectabilis*) in Western Greenland. Artic 52: 188–203.

[60] Petersen MR, Larned WW, Douglas DC (1999) At-sea distribution of Spectacled Eiders (*Somateria fischeri*): 120 year-old mystery resolved. Auk 116: 1009–1020.

[61] Williams TD (2012) Hormones, life-history, and phenotypic variation: Opportunities in evolutionary avian endocrinology. Gen Comp Endocrinol 176: 286–295. 22154573 10.1016/j.ygcen.2011.11.028

[62] Payne RB (1972) Mechanisms and control of molt. In: Farner DS, King JR, editors. Avian biology. Volume 2. New York: Academic Press. pp. 104–155.

[63] Palmer RS (1972) Patterns of molting. In: Farner DS, King JR, editors. Avian Biology, Volume 2. New York: Academic Press. pp. 65–102.

[64] Van De Wetering D, Cooke F (2000) Body weight and feather growth of male Barrow's goldeneye during wing molt. Condor 102(1): 228–231.

[65] Robertson GJ, Savard J-PL (2002) Long-tailed duck (*Clangula hyemalis*). In: Poole A, Gill F, editors. The birds of North America, No 651. Philadelphia PA: The Birds of North America, Inc. 28 pp.

[66] Serra L, Underhill L (2006) The regulation of primary molt speed in the grey plover, Pluvialis squatarola. Acta Zool sinica 52: 451–455.

[67] Dawson A (2004) The effects of delaying the start of moult on the duration of moult, primary feather growth rates and feather mass in Common Starlings *Sturnus vulgaris* . Ibis 146: 493–500.

[68] Serra L (2001) Duration of primary moult affects primary quality in Grey Plovers *Pluvialis squatarola* . J Avian Biol 32: 377–380.

[69] Griggio M, Serra L, Licheri D, Campomori C, Pilastro A (2009) Moult speed affects structural feather ornaments in the Blue Tit. J Evol Biol 22: 782–792. 19320797 10.1111/j.1420-9101.2009.01700.x

[70] Rudebeck G (1950) The choice of prey and modes of hunting of predatory birds with special reference to their selective effect. Oikos 2: 65–88.

[71] Hedenström A, Sunada S (1999) On the aerodynamics of moult gaps in birds. J Exp Biol 202: 67–76. 9841896 10.1242/jeb.202.1.67

[72] Swaddle JP, Williams EV, Rayner M (1999) Effect of simulated flight feather moult on escape take-off performance in starlings. J Avian Biol 30: 351–358.

[73] Portugal SJ, Green JA, Butler PJ (2007) Annual changes in body mass and resting metabolism in captive barnacle geese (*Branta leucopsis*): the importance of wing moult. J Exp Biol 210(8): 1391–1397. 17401121 10.1242/jeb.004598

[74] Fox AD, King R (2011) Body mass loss amongst moulting pochard *Aythya ferina* and tufted duck *A. fuligula* at Abberton Reservoir, south east England. J Ornithol 152: 727–732.

[75] Sjöberg K (1986) The flightless period of free-living male Teal *Anas crecca* in northern Sweden. Ibis 130: 164–171.

[76] Howell MD (2002) Molt dynamics of male long-tailed ducks on the Beaufort Sea. Master Thesis, Auburn University, Auburn, Alabama, USA.

[77] Ouellet J-F, Guillemette M, Blier PU (2008) Morphological and physiological aspects of takeoff aptitudes of female common eiders (*Somateria mollissima*) during the pre-laying period. Can J Zool 86: 462–469 10.1139/Z08-021

